# Supplementary material for: The Developmental Eye Movement Test Does Not Detect Oculomotor Problems: Evidence from Children with Nystagmus
Source: Optom Vis Sci. 2022 Aug 2;99(9):711–7. doi: 10.1097/OPX.0000000000001930 (PMC9470044; doi:10.1097/OPX.0000000000001930)
Supplement: SUPPLEMENTARY MATERIAL [file ovs-99-711-s001.docx]

ORIGINAL INVESTIGATION

The Developmental Eye Movement Test Does Not Detect Oculomotor Problems: Evidence from Children with Nystagmus

Nouk Tanke, PhD, Annemiek D. Barsingerhorn, PhD, Jeroen Goossens, PhD, and F. Nienke Boonstra, MD, PhD

Donders Institute for Brain, Cognition and Behavior, Department of Cognitive Neuroscience, Radboud University Medical Centre, Nijmegen, The Netherlands (NT, ADB, JG, FNB), Donders Institute for Brain, Cognition and Behavior, Department of Biophysics, Radboud University Medical Centre, Nijmegen, The Netherlands (ADB), Royal Dutch Visio, National Foundation for the Visually Impaired and Blind, Nijmegen, The Netherlands (FNB), and Behavioral Science Institute, Radboud University, Nijmegen, The Netherlands (FNB)

Short title: DEM Test Performance with Nystagmus

no tables; 3 figures; 3 appendices

Submitted: December 22, 2021; accepted July 3, 2022.

Corresponding author:

Nienke Boonstra

NienkeBoonstra@visio.org

**ABSTRACT**

Significance: The Developmental Eye Movement (DEM) test, a test purported to assess oculomotor skills, does not detect eye movement disorder in Nystagmus Syndromes. The test should not be used for the clinical evaluation of oculomotor disorders. Purpose: The DEM test ratio compares a horizontal number naming subtest to a vertical one to identify oculomotor problems independent of a child’s visual-verbal naming skills. Here, we tested construct validity of this method by comparing scores of children with and without pathologic nystagmus. Such a nystagmus disturbs normal fixation and saccadic behavior due to the presence of involuntary rhythmic oscillations of the eyes. Therefore, if the ratio is indeed a comprehensive measure of oculomotor problems, children with nystagmus should show an increased ratio score. Methods: The DEM test performances of normally sighted children (n = 94), children with ocular visual impairments (VI_o_, n = 33) and children with cerebral visual impairment (CVI, n = 30) were analyzed using linear regression. Part of the children with VI_o_ and CVI had either Fusion Maldevelopment Nystagmus Syndrome (n = 8) or Infantile Nystagmus Syndrome (n = 20) whereas the others showed no pathologic nystagmus. Results: The times needed for the horizontal and vertical subtests were significantly different between children with normal vision, VI_o_, and CVI (*P* < .001). However, the presence of nystagmus did not add significantly to the horizontal and vertical times (*P* > .2), nor did it have an effect on the ratio (*P* > .1). Conclusions: The DEM test ratio is not sensitive to fixation and saccade abnormalities associated with nystagmus, indicating that it does not have general construct validity to detect true eye movement disorders. While not suitable for the evaluation of oculomotor disorders, the subtests do have clinical relevance in the diagnosis of CVI.

The Developmental Eye Movement test is a commonly used number-naming test to assess and quantify oculomotor skills of children in a simulated reading environment.^1,2^ In contrast to earlier oculomotor assessment tools of visual-verbal format, such as the King-Devick test, ^3, 4^ the Developmental Eye Movement test aims to factor out the effects of rapid automaticity naming skills by including a vertical subtest (Figure 1).^1 5^ The vertical subtest consists of two parts. In each part, two vertical columns of 20 equally spaced numbers are read from top to bottom. In the horizontal subtest, 16 horizontal rows of five unequally spaced numbers are read from left to right. The subtests are scored by completion time and naming errors. Scores for the vertical subtest combine the results of the two vertical arrays. Visual acuity, sustained visual attention, number recognition and retrieval, visual-verbal integration time are but a few factors other than oculomotor skills that influence the results of either subtest. The premise of the Developmental Eye Movement test is, however, that the vertical subtest is dominated by visual-verbal number naming skills (automaticity) rather than oculomotor skills because there is no need for horizontal saccades in this subtest.^1^ Automaticity also influences the time to complete the horizontal subtest, but the test assumes that a higher level of oculomotor control is required for making horizontal saccades of varying magnitude. Therefore, deficiencies in oculomotor performance during the horizontal subtest would increase the discrepancy with the vertical subtest as expressed by the ratio. The Developmental Eye Movement test ratio is defined as the horizontal time (corrected for omission and addition errors) divided by the vertical time. The ratio is the main outcome measure to evaluate oculomotor function.^1^ A ratio higher than the norm would indicate oculomotor dysfunction independent of a child’s rapid automaticity naming skills.^6^

Thus far, however, construct validity of the test ratio remains unclear. Although the horizontal and vertical subtests appear to be good indicators of the level of academic performance,^1,7,8^ reading rate,^9-13^ and speed of visual processing,^14,15^ the assumption that the ratio is a good measure of oculomotor function and symptomatology has often been questioned.^14,16-21^ We have previously measured eye movements while children partook in the Developmental Eye Movement test. Our results showed that, both during the vertical and horizontal subtests, children spent relatively little time making saccades compared to the time spent fixating on numbers.^15^ We therefore argued that the vertical and horizontal subtests on their own are useful to detect delayed visual processing or number naming, but that the ratio is not a good indicator of saccade behavior. In clinical practice, however, oculomotor dysfunction is often defined as a deficiency in the overall process of reading eye movements rather than a deficiency in the execution of saccades alone.^5^ This includes fixation behavior and planning of saccade series.

Our previous eye-tracking experiments^15^ only included normally sighted children who did not have difficulties in oculomotor behavior. A valid question is therefore: how do children with impaired oculomotor functions perform on the Developmental Eye Movement test compared to normally sighted children? Surprisingly, to the best of our knowledge, only few studies have explored this in various clinical cohorts. One study tested children with amblyopia, where one of the symptoms is a poorer efficiency in oculomotor control.^19^ A second study tested a group of children diagnosed with a variety of impaired oculomotor skills.^22^ A recent study tested children with a developmental coordination disorder where pursuit tracking tasks can be one of the challenges they experience.^23^ All three studies found significant associations of the Developmental Eye Movement subtests with visual skills, but no significant relation with oculomotor symptomatology. A fourth study, on the other hand, reported that failing the ratio identified 90% of the children with one or more positive scores on a questionnaire of symptoms associated with oculomotor dysfunction.^24^

In the present paper, we compared data from children with and without clinical signs of abnormal oculomotor behavior to study whether the Developmental Eye Movement test ratio can differentiate between problems with seeing, decoding, and rapidly verbalizing the numbers on the one hand, and difficulty in oculomotor control on the other. More specifically, we focused on children whose fixation and saccadic behavior was disturbed by the presence of a pathologic nystagmus. Nystagmus may be defined as repetitive, to-and-fro involuntary eye movements. It commonly consists of an alternation of slow drift (slow phase) in one direction and corrective saccade (quick phase) in the other.^25^ Even if its etiology and waveform may differ between individuals,^26^ the involuntary drifts and quick-phases of a pathologic nystagmus are abnormal forms of oculomotor behavior causing abnormal patterns of fixations and saccades during reading and non-reading tasks. These abnormal patterns are the “fixational and saccadic activity”, which the Developmental Eye Movement test aims to quantify.^6^

Infantile nystagmus can often be observed through visual inspection,^27^ and is associated with reduced visual acuity (for a review, see Papageorgiou et al, 2014^28^). Infantile nystagmus is congenital or acquired in the first twelve months of life. The cause can be unknown (idiopathic) or associated with ocular disease or neurological syndromes.^29^ Nystagmus can manifest along different planes, waveforms, amplitude and conjugacy, but all variations are characterized as oculomotor deficiencies. ^30^ Fusion Maldevelopment Nystagmus Syndrome is characterized as a jerk nystagmus that primarily occurs when one eye is closed. ^31^ People with infantile nystagmus often show longer saccade latencies than people without nystagmus and saccade accuracy is reduced.^32-36^

Infantile nystagmus is seen in children with ocular visual impairments and symptomatically in children with cerebral visual impairments.^37^ Children with cerebral visual impairments suffer from cognitive visual impairments caused by malfunctions in the visual pathways of the brain.^38^ They show a large variation in symptoms, visual acuity and cerebral damage, while obvious ocular abnormalities are not found.^39-41^ Their visual orienting behavior consists of fixation abnormalities, such as a prolonged time before fixating on a stimulus and intermittent fixation towards a stimulus.^42,43^ Additionally, they show oculomotor abnormalities; nystagmus and strabismus are often found as a symptom of cerebral visual impairment, also when their family history is negative for strabismus or nystagmus.^37^ We have recently shown that children with cerebral visual impairments needed more time to read the numbers of the Developmental Eye Movement subtests than children with ocular visual impairments. Additionally, the children with ocular visual impairments or cerebral visual impairments needed, on average, more time than the normally sighted controls.^44^ Given the abnormal visual orienting behaviors of children with cerebral visual impairments, we wondered if the ratio could be informative too.

If the Developmental Eye Movement test ratio is a good, comprehensive indicator for oculomotor function, then children with ocular visual impairments or cerebral visual impairments who also have a clinically visible nystagmus should show particularly high ratios compared to age-matched children without nystagmus, be it normally sighted children or children with visual impairments. For children with cerebral visual impairments, one might perhaps expect a higher-than-normal ratio even if they have no nystagmus because the spatial and temporal information from visual cortex may be delayed or not accessible for efficient planning and execution of visually guided eye movements.^45,46^

**METHODS**

**Participants**

This dataset and part of the methods were previously described.^44^ A total number of 157 children were included. Children who did not show signs of nystagmus (n = 129, 9.4 ± 1.9 years), consisted of three different groups: normally sighted children (n =94), children with ocular visual impairments (n = 13) and children with cerebral visual impairments (n = 22). Children with nystagmus were subdivided into children with Infantile Nystagmus Syndrome (n = 20, 9.5 ± 2.6 years, ocular visual impairments; n = 18, cerebral visual impairments; n = 2), and children with Fusion Maldevelopment Nystagmus Syndrome (n = 8, 8.1 ± 1.1 years, ocular visual impairments; n = 2, cerebral visual impairments; n = 6).

Five children with ocular visual impairments and nystagmus had albinism (see ^44^ for more detail). The normally sighted children had a distance visual acuity of 0.1 logMAR or better (mean = -0.23 ± 0.08 logMAR), whereas the children with ocular visual impairments had a visual acuity worse than 0.1 logMAR (mean = 0.38 ± 0.23 logMAR). Inclusion criteria for normally sighted children and children with ocular visual impairments were: normal birth weight (>2500 g), birth at term (>36 weeks), no perinatal complications and normal development. The only inclusion criterium for the children with cerebral visual impairments was having the diagnosis of cerebral visual impairments (mean visual acuity = 0.17 ± 0.25 logMAR).

The diagnosis of cerebral visual impairments as well as the presence of nystagmus was determined by ophthalmologists of Bartiméus or Royal Dutch Visio, Dutch institutes for the rehabilitation of the visually impaired. Cerebral visual impairment was diagnosed according to the Dutch guidelines for cerebral visual impairment, which takes into consideration medical history and ophthalmological, neuropsychological and neurological examinations.^47^ Nystagmus was determined during ophthalmological examination. In case of doubt, slit lamp examination was used.

The study was approved by the local ethics committee (Commissie Mensgebonden Onderzoek regio Arnhem-Nijmegen, The Netherlands. Protocol NL48708.091.14), and conducted according to the principles of the Declaration of Helsinki. Informed consent was obtained from the parents of all participants before testing. Testing occurred at the children’s primary school (normally sighted children) or rehabilitation center (children with visual impairments) from which they were recruited.

**Developmental Eye Movement test**

The Developmental Eye Movement test (Figure 1) was administered on a computer screen at ~65 cm, with all numbers presented at the prescribed^48^ font size of 0.71 logMAR. Before testing, the children first practiced in a shortened version of the Developmental Eye Movement test with random ordering of the numbers to familiarize them with the task and make sure that they could read numbers. Then, in Test A, children had to name the numbers from top to bottom, one column at a time. Test A was followed by test B, which is like test A but with the numbers in a different order. Lastly, in test C, the children were asked to name the numbers from left to right, starting at the top row. For the full list of numbers used and details concerning number spacing and number size, see ^1, 15^. In each subtest, the array of numbers appeared on the screen as soon as the experimenter pressed the space bar, and disappeared when the experimenter pressed the space bar again as soon as the child named the last number. The software recorded the start and stop moments.

**Equipment**

The digital version of the Developmental Eye Movement test used at the schools and Bartiméus was written in Matlab (version 2013b, Mathworks, Natick, MA) using the Psychophysics Toolbox (version 3.0.12, Mathworks, Natick, MA).^49^ The one used at Royal Dutch Visio was written in Python using PsychoPy3 (version 2020.2.10, Open Science Tools, Ltd., Nottingham, UK).^50^ In all cases, the visual stimuli were presented on a 23-inch LCD screen (Dell, Inc. 1920x1200 pixels, Round Rock, TX).

**Data Analyses**

The data were plotted and analyzed in Matlab (version 2020b, Mathworks, Natick, MA). Vertical time was taken as the sum of the time needed to complete test A time and test B. If only test A was completed (n = 4/157), vertical time was taken as 2 * test A. ^15^ Horizontal time was the time to complete test C adjusted for omissions and additions.^48^ Repeating a whole line counted as five addition errors. Skipping one-line counted as two omission errors.

$$Horizontal time=Test C time\times\frac{80}{(80-omissions+additions)}$$

In accordance with the scoring rules of the Developmental Eye Movement test, ^48^ the time needed for test A and B was not adjusted for errors (The number of errors made during these tests is indeed small). The ratio was determined as horizontal time (adjusted for errors) divided by vertical time:

$$ratio=\frac{horizontal time}{vertical time}$$

We then used multiple linear regression to test if the presence of nystagmus influences the test scores. In these analyses, we divided the population into three different groups: normally sighted children, children with ocular visual impairments and children diagnosed with cerebral visual impairment. In addition, we categorized the children according to the presence of nystagmus using three different levels: no clinical signs of nystagmus, Fusion Maldevelopment Nystagmus Syndrome and Infantile Nystagmus Syndrome. Since the test scores improved with age, we also included age as a covariate. The regression model applied to the horizontal time, vertical time and ratio was as follows (Wilkinson notation): *DEM score ~ nystagmus + group + age.*

While most of the children performed the Developmental Eye Movement subtests as instructed, the horizontal subtest was too difficult for eight of them (Infantile Nystagmus Syndrome; n = 0/20, Fusion Maldevelopment Nystagmus Syndrome; n = 2/8, no nystagmus; n = 6/129). These children did not read the numbers row by row, but skipped from one row to another on numerous occasions, making it impossible for the experimenter to document which number was read from which location. We therefore had to exclude the horizontal times and test ratios of this small number of children (8/157).

**RESULTS**

**Horizontal and Vertical Performance**

The tested population consisted of 94 normally sighted children who did not have nystagmus, and 63 children with ocular visual impairments or cerebral visual impairments of whom 28 showed a pathologic nystagmus. Figures 2A-B show that the children’s horizontal and vertical times improved as a function of their calendar age. Moreover, it can be seen that compared to age-matched normally sighted children (black dots), most of the children with ocular visual impairments or cerebral visual impairments needed more time to read the numbers of the Developmental Eye Movement test. However, in children diagnosed with ocular visual impairments or cerebral visual impairments there appeared to be no systematic differences in Developmental Eye Movement performance between children with Infantile Nystagmus Syndrome (red dots), children with Fusion Maldevelopment Nystagmus Syndrome (blue dots) and children without nystagmus (gray dots).

Our multiple linear regression analyses confirmed our previous findings ^44^ that: 1) the children’s age and the diagnosis ocular visual impairments or cerebral visual impairments had a significant influence on both the vertical times (age: *t*(151) = -6.32, ***P*** < .001; ocular visual impairments: *t*(151) = 3.32, ***P*** = .001; cerebral visual impairments: *t*(151) = 5.83, ***P*** < .001) and the horizontal times (age: t(143) = -7.87, ***P*** < .001; ocular visual impairments: *t*(143) = 2.17, ***P*** = .03; cerebral visual impairments: *t*(143) = 6.18, ***P*** < .001), ^44^ and that 2) children with cerebral visual impairments needed more time for the horizontal subtest than children with ocular visual impairments (cerebral visual impairments: 21 ± 8 ms longer, *t*(143) = 2.76, ***P*** = .007). They also confirmed our new observation that the presence of a pathologic nystagmus had no significant influence on performance in either the vertical subtest (Fusion Maldevelopment Nystagmus Syndrome: t(151) = 0.99, ***P*** = .32, Infantile Nystagmus Syndrome: *t*(151) = -1.26, ***P*** = .21) or the horizontal subtest (Fusion Maldevelopment Nystagmus Syndrome: *t*(143) = -0.37, ***P*** = .71, Infantile Nystagmus Syndrome: *t*(143) = -0.53, ***P*** = .60, linear regression). To illustrate these findings, figure 2C shows the average differences of children with Fusion Maldevelopment Nystagmus Syndrome (blue) or Infantile Nystagmus Syndrome (red) compared to children without nystagmus adjusted for the effects of age and diagnosis ocular visual impairments or cerebral visual impairments. Appendix Tables A1A-B, available at http://links.lww.com/OPX/A576, lists the ANOVA tables for these analyses.

**Results for Children with Nystagmus**

The Developmental Eye Movement test ratio was purported to differentiate between poor oculomotor skills (assumed to increase horizontal time and ratio) and poor visual-verbal automaticity (horizontal and vertical times would both increase, but the ratio would remain normal).^1^ However, as can be seen in figure 3, the mean difference in ratio between children with or without nystagmus is nearly zero. Compared to age-matched normally sighted children, the average ratio of children with Fusion Maldevelopment Nystagmus Syndrome and Infantile Nystagmus Syndrome was only 0.02 ± 0.10 and 0.03 ± 0.07 larger, respectively. Thus, we found no significant relationship between the ratio and the presence of nystagmus (Fusion Maldevelopment Nystagmus Syndrome: *t*(143) = 0.17, ***P*** = .86; Infantile Nystagmus Syndrome: *t*(143) = 0.41, ***P*** = .68; linear regression). Additionally, the ratio was not significantly influenced by the diagnosis ocular visual impairments or cerebral visual impairments (ocular visual impairments: *t*(143) = -0.95, ***P*** = .34; cerebral visual impairments: *t*(143) = 1.36, ***P*** = .17). The ratio only showed an age-related decrease (-0.022 ± 0.009 per year; *t*(143) = -2.46, ***P*** = .02), as was also reported by Garzia et al ^1^. See Appendix Table A1C, available at http://links.lww.com/OPX/A576, for the ANOVA table for this analysis.

**DISCUSSION**

Both the horizontal and vertical times as well as the ratio were similar for children with or without clinically apparent nystagmus. The presence of an ocular visual impairment or cerebral visual impairment was not significantly reflected in the ratio either. Children diagnosed with an ocular visual impairment or cerebral visual impairment did perform significantly worse on the horizontal and vertical tests than age-matched normally sighted children.

Note that a large group of normally sighted children was included (94/157), and none of them had nystagmus. We have previously reported eye-tracking data from these children during the horizontal and vertical subtest. ^15^ Unfortunately, we did not have sufficient eye-tracking data from the children with ocular visual impairments and cerebral visual impairments (but see Appendix Figures A1 and A2, available at http://links.lww.com/OPX/A576, for three illustrative cases). In the present study, we therefore had to rely on the status reports of the patients regarding their oculomotor deficiencies. Twenty children in our study were reported to have Infantile Nystagmus Syndrome. For these children, the fixation and saccade abnormalities associated with their nystagmus should have been reflected in an abnormally high ratio. However, this was not observed. An additional eight children with Fusion Maldevelopment Nystagmus Syndrome were identified. In Fusion Maldevelopment Nystagmus Syndrome, the involuntary oscillations of the eyes typically occur only when one eye is covered. Since the Developmental Eye Movement test was performed binocularly, the children with Fusion Maldevelopment Nystagmus Syndrome most likely did not exhibit nystagmus during the test. Thus, one would not necessarily expect abnormal fixation and saccadic behavior for these children. Some children with Fusion Maldevelopment Nystagmus Syndrome might have been misdiagnosed: they were not subjected to detailed eye movement recordings to confirm that the nystagmus was absent during the Developmental Eye Movement test. ^51^ To make sure that our results would not be biased by this subgroup of children, we modelled them as a separate category in our regression analyses.

The children with nystagmus were all visually impaired due to ocular problems or they were diagnosed with cerebral visual impairments. On average, children with ocular visual impairments or cerebral visual impairments needed more time to read the numbers of the Developmental Eye Movement subtests than normally sighted controls. We cannot exclude that the children with ocular visual impairments or cerebral visual impairments who showed no signs of nystagmus had other more covert oculomotor deficiencies. Therefore, one should not conclude that the difference in test performance between normally sighted children and children with ocular visual impairments or cerebral visual impairments were exclusively due to visual deficiencies or poorer number naming skills. In subjects with normal development, compensating head movement are often seen (also vertically), while in children with cerebral visual impairment, the coordination between head and eye movements can be impaired too.

The lack of differences in Developmental Eye Movement test performance between children with or without nystagmus is consistent with previous studies showing that reading speed can be nearly normal for people with infantile nystagmus^52^ if an optimal font size is used^53^ and crowding is limited.^54^ While the Developmental Eye Movement subtests can be a good indicator of reading rate,^9-13^ the numbers of the test are large enough for the majority of people with nystagmus to read without limitations ^53^ and are spaced too far apart to be considered crowded.^55^

Most importantly, the ratio, which is supposed to be the key metric of oculomotor performance during the test, was not able to predict the presence of either Fusion Maldevelopment Nystagmus Syndrome or Infantile Nystagmus Syndrome. Together with the fact that the ratio did not differ significantly between normally sighted children, children with ocular visual impairments, or children with cerebral visual impairments, this is a strong indication that the ratio is not sensitive enough to be a clinically relevant diagnostic aid. We suspect that the assumptions underlying this test metric are incorrect. One implicit, but likely invalid, assumption is that children, who exhibit inefficient eye movement patterns in the horizontal subtest, would have no problems planning and executing saccades the vertical subtest. At least in children with a clinically visible nystagmus, this assumption is not tenable. Even if the nystagmus is primarily horizontal, it may still interfere with the planning and execution of a next voluntary saccade because the amplitude and direction of that saccade might need constant adjustment (see Appendix Figure A1, available at http://links.lww.com/OPX/A576, for an example). In children with cerebral visual impairments, whose planning and execution of visually guided eye movements may be impaired due to damage or dysfunction of striate and extra-striate cortex,^38-41^ it is also improbable that the resulting oculomotor dysfunction would differentially affect the two subtests. The neurophysiological organization of the saccadic system at the cortical and subcortical level does not justify this assumption.^25,56,57^

A small number of children (8/157) were excluded from our analyses because the horizontal subtest was too difficult for them. Note, however, that most of these children (6/8) did not show signs of nystagmus (Methods). If anything, the opposite would have been expected if the comparison between the horizontal and vertical subtest were to indicate an oculomotor deficiency.

In conclusion, we believe that the vertical and horizontal subtests are useful in the diagnosis of cerebral visual impairments and in the detection of delayed visual processing speed or number naming skills. However, we found no evidence that the ratio has predictive power concerning visual impairment or the presence of involuntary drifts and saccades.

**ACKNOWLEDGMENTS**

The authors thank the children and their parents for participating, the schools and Bartimeus Sonneheerdt for the use of their examination rooms, and the staff at Royal Dutch Visio in Nijmegen and Rotterdam for sharing their expertise.

**APPENDICES**

**Appendix Table A1**, available at http://links.lww.com/OPX/A576. ANOVA tables of the multiple linear regression analyses for the Developmental Eye Movement (DEM) test. Nystagmus had no significant influence on the vertical subtest **(A)**, horizontal subtest **(B)** or DEM test ratio **(C)** while age did. Diagnosis ocular visual impairment or cerebral visual impairment influenced the outcome of the vertical and horizontal subtest but not the ratio.

**Appendix Figure A1**, available at http://links.lww.com/OPX/A576. Eye movements during the DEM test in two visually impaired children. Although we did not collect sufficient eye-tracking data during the Developmental Eye Movement test to allow for an analysis of the eye movement patterns in children with ocular or cerebral visual impairments, we did obtain some data from a few illustrative cases. **(A)** Eye movements of a child (age: 11.9 years, visual acuity: 0.27 logMAR, left eye) with ocular visual impairments and Infantile Nystagmus Syndrome illustrating how the nystagmus affected the child’s fixation and saccadic behavior in the vertical and horizontal subtests. Top left: point of gaze on the screen (blue) during part of the vertical and horizontal test subtest. Numbers not drawn to scale. Top right: horizontal (red) and vertical (blue) eye position as a function of time during part of the vertical subtest. Bottom: eye position traces during part of the horizontal subtest. **(B)** Same as **(A)** but from a child (age: 8.6 years, visual acuity: 0.14 logMAR, left eye) having no nystagmus but a lot of difficulty in both subtests. The difficulties in completing both subtests were congruent with the child’s diagnosis of cerebral visual impairment. Note the many saccades back up during the vertical subtest, and the child’s difficulty localizing the numbers of the horizontal subtest (see Appendix Figure A3 for comparison). Eye movement data were collected with a remote, stereoscopic eye tracker under head-free viewing conditions. ^15, 58^

**Appendix Figure A2**, available at http://links.lww.com/OPX/A576. Eye movements during the Developmental Eye Movement test in a normally sighted child. The child was 10.5 years old, visual acuity was -0.15 logMAR, left eye is shown. **(A-B)** Point of gaze on the screen (blue) during the vertical **(A)** and horizontal **(B)** subtest. Circles represent number locations. **(C-D)** Horizontal (red) and vertical (blue) eye positions a function of time during the vertical **(C)** and the horizontal subtest **(D).** Data were collected under head-free viewing conditions. Eye positions (in **C** and **D**) are eye positions relative to the head. Blinks and other eye-tracking artifacts were removed.

**REFERENCES**

1. Garzia RP, Richman JE, Nicholson SB, Gaines CS. A New Visual-Verbal Saccade Test: The Development Eye Movement Test (DEM). J Am Optom Assoc 1990;61:124-35.

2. Richman JE, Walker AJ, Garzia RP. The Impact of Automatic Digit Naming Ability on a Clinical Test of Eye Movement Functioning. J Am Optom Assoc 1983;54:617-22.

3. King AT, Devick S. The Proposed King-Devick Test and Its Relation to the Pierce Saccade Test and Reading Levels [Senior Research Project. Illinois College of Optometry; 1976.

4. Lieberman S, Cohen AH, Rubin J. Nysoa K-D Test. J Am Optom Assoc 1983;54:631-7.

5. Facchin A. Spotlight on the Developmental Eye Movement (DEM) Test. Clin Optom (Auckl) 2021;13:73-81.

6. Richman JE. Developmental Eye Movement Test, Examiner’s Manual, Version 2.0. South Bend, IN: Bernell Corp.; 2009.

7. Hopkins S, Black AA, White SL, Wood JM. Visual Information Processing Skills Are Associated with Academic Performance in Grade 2 School Children. Acta Ophthalmol 2019;97:e1141-e8.

8. Wood JM, Black AA, Hopkins S, White SL. Vision and Academic Performance in Primary School Children. Ophthalmic Physiol Opt 2018;38:516-24.

9. Palomo-Alvarez C, Puell MC. Relationship between Oculomotor Scanning Determined by the Dem Test and a Contextual Reading Test in Schoolchildren with Reading Difficulties. Graefes Arch Clin Exp Ophthalmol 2009;247:1243-9.

10. Northway N. Predicting the Continued Use of Overlays in School Children--a Comparison of the Developmental Eye Movement Test and the Rate of Reading Test. Ophthalmic Physiol Opt 2003;23:457-64.

11. Facchin A, Maffioletti S, Carnevali T. Validity Reassessment of Developmental Eye Movement (DEM) Test in the Italian Population. Optom Vis Dev 2011;42:155-67.

12. Serdjukova J, Ekimane L, Valeinis J, et al. How Strong and Weak Readers Perform on the Developmental Eye Movement Test (DEM): Norms for Latvian School-Aged Children. Read Writ 2016;30:233-52.

13. Vernet M, Bellocchi S, Leibnitz L, et al. Predicting Future Poor Readers from Pre-Reading Visual Skills: A Longitudinal Study. Appl Neuropsychol Child 2021:1-15.

14. Ayton LN, Abel LA, Fricke TR, McBrien NA. Developmental Eye Movement Test: What Is It Really Measuring? Optom Vis Sci 2009;86:722-30.

15. Tanke N, Barsingerhorn AD, Boonstra FN, Goossens J. Visual Fixations Rather Than Saccades Dominate the Developmental Eye Movement Test. Sci Rep 2021;11:1162.

16. Facchin A, Maffioletti S. The Reliability of the DEM Test in the Clinical Environment. Front Psychol 2018;9:1279.

17. Orlansky G, Hopkins KB, Mitchell GL, et al. Reliability of the Developmental Eye Movement Test. Optom Vis Sci 2011;88:1507-19.

18. Rouse MW, Nestor EM, Parot CJ, Deland PN. A Reevaluation of the Developmental Eye Movement (DEM) Test's Repeatability. Optom Vis Sci 2004;81:934-8.

19. Webber AL, Wood JM, Gole GA, Brown B. Effect of Amblyopia on the Developmental Eye Movement Test in Children. Optom Vis Sci 2009;86:760-6.

20. Moiroud L, Gerard CL, Peyre H, Bucci MP. Developmental Eye Movement Test and Dyslexic Children: A Pilot Study with Eye Movement Recordings. PLoS One 2018;13:e0200907.

21. Medland C, Walter H, Woodhouse JM. Eye Movements and Poor Reading: Does the Developmental Eye Movement Test Measure Cause or Effect? Ophthalmic Physiol Opt 2010;30:740-7.

22. Kulp MT, Schmidt PP. Relationship between Visual Skills and Performance on Saccadic Eye Movement Testing. Optom Vis Sci 1998;75:284-7.

23. Bellocchi S, Ducrot S, Tallet J, et al. Effect of Comorbid Developmental Dyslexia on Oculomotor Behavior in Children with Developmental Coordination Disorder: A Study with the Developmental Eye Movement Test. Hum Mov Sci 2021;76:102764.

24. Tassinari JT, DeLand P. Developmental Eye Movement Test: Reliability and Symptomatology. Optometry 2005;76:387-99.

25. Leigh RJ, Zee DS. The Neurology of Eye Movements. Oxford, UK: Oxford University Press; 2015.

26. Dell'Osso LF, Daroff RB. Congenital Nystagmus Waveforms and Foveation Strategy. Doc Ophthalmol 1975;39:155-82.

27. Serra A, Leigh RJ. Diagnostic Value of Nystagmus: Spontaneous and Induced Ocular Oscillations. J Neurol Neurosurg Psychiatry 2002;73:615-8.

28. Papageorgiou E, McLean RJ, Gottlob I. Nystagmus in Childhood. Pediatr Neonatol 2014;55:341-51.

29. Dell'Osso LF, Hertle RW, Daroff RB. "Sensory" and "Motor" Nystagmus: Erroneous and Misleading Terminology Based on Misinterpretation of David Cogan's Observations. Arch Ophthalmol 2007;125:1559-61.

30. Proudlock FA, Gottlob I. In: Taylor D, Hoyt CS, eds. Pediatric Opththalmology and Strabismus, 4^th^ ed. Kidlington, UK: Elsevier B.V.; 2011.

31. American Academy of Opthalmology (AAO). Dell'Osso LF. Nystagmus in Infancy and Childhood; 2018. Available at: <https://www.aao.org/disease-review/nystagmus-in-infancy-childhood> . Accessed: February 15, 2022.

32. Dunn MJ, Margrain TH, Woodhouse JM, Erichsen JT. Visual Processing in Infantile Nystagmus Is Not Slow. Invest Ophthalmol Vis Sci 2015;56:5094-101.

33. Huurneman B, Boonstra FN, Goossens J. Perceptual Learning in Children with Infantile Nystagmus: Effects on Visual Performance. Invest Ophthalmol Vis Sci 2016;57:4216-28.

34. Pel JJ, Kooiker MJ, van der Does JM, et al. Orienting Responses to Various Visual Stimuli in Children with Visual Processing Impairments or Infantile Nystagmus Syndrome. J Child Neurol 2014;29:1632-7.

35. Wang ZI, Dell'Osso LF. Being "Slow to See" Is a Dynamic Visual Function Consequence of Infantile Nystagmus Syndrome: Model Predictions and Patient Data Identify Stimulus Timing as Its Cause. Vision Res 2007;47:1550-60.

36. Worfolk R, Abadi RV. Quick Phase Programming and Saccadic Re-Orientation in Congenital Nystagmus. Vision Res 1991;31:1819-30.

37. Bosch DG, Boonstra FN, Willemsen MA, et al. Low Vision Due to Cerebral Visual Impairment: Differentiating between Acquired and Genetic Causes. BMC Ophthalmol 2014;14:59.

38. Dutton GN, Jacobson LK. Cerebral Visual Impairment in Children. Semin Neonatol 2001;6:477-85.

39. Philip SS, Dutton GN. Identifying and Characterising Cerebral Visual Impairment in Children: A Review. Clin Exp Optom 2014;97:196-208.

40. Lueck AH, Dutton GN, Chokron S. Profiling Children with Cerebral Visual Impairment Using Multiple Methods of Assessment to Aid in Differential Diagnosis. Semin Pediatr Neurol 2019;31:5-14.

41. Pehere N, Chougule P, Dutton GN. Cerebral Visual Impairment in Children: Causes and Associated Ophthalmological Problems. Indian J Ophthalmol 2018;66:812-5.

42. Pel J, Does LV, Boot F, et al. Effects of Visual Processing and Congenital Nystagmus on Visually Guided Ocular Motor Behaviour. Dev Med Child Neurol 2011;53:344-9.

43. Porro G, Dekker EM, Van Nieuwenhuizen O, et al. Visual Behaviours of Neurologically Impaired Children with Cerebral Visual Impairment: An Ethological Study. Br J Ophthalmol 1998;82:1231-5.

44. Tanke N, Barsingerhorn AD, Goossens J, Boonstra FN. The Developmental Eye Movement Test as a Diagnostic Aid in Cerebral Visual Impairment. Front Hum Neurosci 2021;15:732927.

45. Good WV, Jan JE, Burden SK, et al. Recent Advances in Cortical Visual Impairment. Dev Med Child Neurol 2001;43:56-60.

46. Kelly JP, Phillips JO, Saneto RP, et al. Cerebral Visual Impairment Characterized by Abnormal Visual Orienting Behavior with Preserved Visual Cortical Activation. Invest Ophthalmol Vis Sci 2021;62:15.

47. Federatie Medisch Specialisten. Cerebral Visual Impairment (CVI); 2019. Available at: <https://richtlijnendatabase.nl/richtlijn/cerebral_visual_impairment_cvi/startpagina_-_cvi.html#algemeen>. Accessed: February 15, 2022.

48. Richman JE, Garzia RP. Developmental Eye Movement Test, Examiner's Booklet, Version 1. South Bend, IN: Bernell Corp.; 1987.

49. Kleiner M, Brainard D, Pelli D, et al. What's New in Psychtoolbox-3. Perception 2007;36:1-16.

50. Peirce J, Gray JR, Simpson S, et al. Psychopy2: Experiments in Behavior Made Easy. Behav Res Methods 2019;51:195-203.

51. Dell'Osso LF, Schmidt D, Daroff RB. Latent, Manifest Latent, and Congenital Nystagmus. Arch Ophthalmol 1979;97:1877-85.

52. Dysli M, Abegg M. Nystagmus Does Not Limit Reading Ability in Albinism. PLoS One 2016;11:e0158815.

53. Barot N, McLean RJ, Gottlob I, Proudlock FA. Reading Performance in Infantile Nystagmus. Ophthalmology 2013;120:1232-8.

54. Huurneman B, Boonstra FN, Goossens J. Perceptual Learning in Children with Infantile Nystagmus: Effects on Reading Performance. Invest Ophthalmol Vis Sci 2016;57:4239-46.

55. Huurneman B, Boonstra FN, Cox RF, et al. A Systematic Review on 'Foveal Crowding' in Visually Impaired Children and Perceptual Learning as a Method to Reduce Crowding. BMC Ophthalmol 2012;12:27.

56. Pierrot-Deseilligny C, Rivaud S, Gaymard B, et al. Cortical Control of Saccades. Ann Neurol 1995;37:557-67.

57. Pouget P. The Cortex Is in Overall Control of 'Voluntary' Eye Movement. Eye (Lond) 2015;29:241-5.

58. Barsingerhorn AD, Boonstra FN, Goossens J. Development and Validation of a High-Speed Stereoscopic Eyetracker. Behav Res Methods 2018;50:2480-97.

**FIGURE LEGENDS**

**Figure 1.** The Developmental Eye Movement (DEM) test. Schematic overview of the DEM test. Numbers not drawn to scale. The vertical subtest must be read from top to bottom and is defined as the time needed to read test A + B. The horizontal subtest has to be read from left to right, and is defined by test C after reading time is corrected for errors.

**Figure 2.** Performance on the vertical and the horizontal Developmental Eye Movement (DEM) test. **(A)** Total time needed to read the numbers of the vertical subtest plotted against age for children with Infantile Nystagmus Syndrome (INS, red dots), Fusion Maldevelopment Nystagmus Syndrome (FMNS, blue dots), normally sighted children (NS) without nystagmus (NS NN, black dots), and children with ocular visual impairments or cerebral visual impairments without nystagmus (VI_o_/CVI NN, gray dots). **(B)** Same as **(A)**, but for the horizontal time. **(C)** Average difference of the score of children with Fusion Maldevelopment Nystagmus Syndrome (FMNS, blue bars) and Infantile Nystagmus Syndrome (INS, red bars) compared to the scores of children with no nystagmus, adjusted for age and group (NS, VI_o_, or CVI). CVI = cerebral visual impairment. Error bars represent ± 1 SEM.

**Figure 3.** The Developmental Eye Movement (DEM) ratio. **(A)** The DEM ratio (horizontal time/vertical time) plotted against age for children with Infantile Nystagmus Syndrome (INS, red dots), Fusion Maldevelopment Nystagmus Syndrome (FMNS, blue dots), normally sighted children (NS) without nystagmus (NS NN, black dots), and children with ocular visual impairments or cerebral visual impairments without nystagmus (VI_o_ /CVI NN, gray dots). **(B)** Average difference of the ratio of children with Fusion Maldevelopment Nystagmus Syndrome (FMNS, blue bars) and Infantile Nystagmus Syndrome (INS, red bars) compared to the ratio of children with no nystagmus, adjusted for age and group (NS, VI_o_, or CVI). CVI = cerebral visual impairment. Error bars represent ± 1 SEM.
